# CRISPR-Cas9 enables efficient genome engineering of the strictly lytic, broad-host-range staphylococcal bacteriophage K

**DOI:** 10.1128/aem.02014-24

**Published:** 2025-08-04

**Authors:** Jonas Fernbach, Jasmin Baggenstos, Ellen-Aleksandra Svorjova, Jeannine Riedo, Shawna McCallin, Martin J. Loessner, Samuel Kilcher

**Affiliations:** 1 Department of Health Science and Technology, ETH Zürich 27219, Zürich, Switzerland; 2 Department of Neuro-Urology, Balgrist University Hospital, University of Zürich 27217, Zürich, Switzerland; University of Nebraska-Lincoln, Lincoln, Nebraska, USA

**Keywords:** *Staphylococcus aureus*, bacteriophage, genetic engineering, antibiotic resistance

## Abstract

*Staphylococcus aureus* is a major opportunistic pathogen, increasingly difficult to treat due to rising resistance to methicillin, vancomycin, and other antimicrobials. Bacteriophages offer a promising alternative, particularly when conventional therapies fail and their efficacy can be enhanced through genetic engineering. Among *S. aureus* phages, the strictly lytic, broad-host-range members of the *Twortvirinae* subfamily are among the most promising therapeutic candidates. However, their large genome sizes make them notoriously difficult to engineer. In this study, we utilized *Twortvirus* K as a model to develop an efficient phage engineering platform, leveraging homologous recombination and CRISPR-Cas9-assisted counterselection. As proof of principle, this platform was utilized to construct a nanoluciferase (*nluc*)-encoding reporter phage (K::*nluc*) and tested as a bioluminescence-based approach for identifying viable *Staphylococcus* cells. Independent of their phage-resistance profile, 100% of tested clinical *S. aureus* isolates emitted bioluminescence upon K::*nluc* challenge. This diagnostic assay was further adapted to complex matrices such as human whole blood and bovine raw milk, simulating *S. aureus* detection scenarios in bacteremia and bovine mastitis. Beyond reporter phage-based diagnostics, our engineering technology opens avenues for the design and engineering of therapeutic *Twortvirinae* phages to combat drug-resistant *S. aureus* strains.

**IMPORTANCE:**

Phage engineering, the process of modifying bacteriophages to enhance or customize their properties, offers significant potential for advancing precision antimicrobial therapies and diagnostics. While methods for engineering small *Staphylococcus* phage genomes are well-established, larger *Staphylococcus* phages have historically been challenging to modify. In this study, we present a novel method that enables the engineering of *Twortvirinae*, a subfamily of *Staphylococcus* phages known for their broad host range and strictly lytic lifestyle, making them highly relevant for diagnostic and therapeutic applications. Using this method, we successfully developed a phage-based diagnostic tool capable of rapid and sensitive detection of *S. aureus* cells across various matrices. This approach has the potential to extend beyond diagnostics, enabling applications such as phage-mediated delivery of antimicrobial effector proteins in the future.

## INTRODUCTION

The prevalence of multi-drug-resistant (MDR) pathogens among the human population has been steadily increasing in recent decades (1–4). The WHO speculates that deaths associated with these MDR organisms might even surpass cancer-related fatalities by 2050 (5). While small-molecule antibiotics have been pivotal in treating widespread diseases, their easy accessibility, over-prescription, and extensive use in medical and agricultural fields have fueled the emergence of resistant strains (1, 6, 7). Bacteriophages and phage-encoded proteins such as endolysins (enzymes that degrade the bacterial cell wall) present promising alternatives to conventional antibiotic treatment. They are being postulated as a significant force in combating the antibiotic resistance crisis in the coming years (8, 9).

Past case reports demonstrated the successful application of phages to treat MDR infections (10, 11). Rapid advances in synthetic biology and genetic engineering have allowed for the design, development, and application of genetically altered phage variants with enhanced clinical potential. Furthermore, novel methods for genetically modifying phages to achieve enhanced functionality are constantly being developed. These innovations in phage therapy are evidenced by recent studies and technological advancements, which are summarized in references 12–15.

Through the strategic selection and engineering of the phage backbone, we can fine-tune crucial characteristics, such as host specificity, to cater to specific clinical applications. These methods have been employed to transition phages to entirely new hosts (16–19), modify the infection cycle for clinical suitability (20), and deliver antimicrobial payload genes directly to the infection site (21). The field of synthetic biology-based engineering is also expanding, and it now encompasses a variety of sophisticated DNA manipulation techniques. These include the isothermal Gibson assembly (22), yeast-based recombineering (23), and whole-genome synthesis, all of which can be utilized for phage genome modification.

*Staphylococcus aureus*, a prevalent opportunistic pathogen, colonizes up to 50% of humans and can result in severe diseases such as pneumonia, respiratory, surgical, and cardiovascular infections, as well as nosocomial bacteremia (24). Bacteremia alone has an estimated annual incidence of up to 50 cases per 100,000, with 10–30% of these patients died as a direct result of the infection (25). Furthermore, resistance emergence in *S. aureus* isolates against a number of different antibiotics has been reported. Methicillin-resistant *S. aureus* (MRSA) and vancomycin-resistant *S. aureus* (VRSA) are two of the variants of greatest concern. According to the CDC’s 2019 report, there were more than 323,700 cases of MRSA and 10,600 attributed deaths in 2017 in the United States alone, although the incidence has been decreasing over the years (26).

Bacteriophages from the *Twortvirinae* subfamily, like the model phage K and its close relatives, are ideal candidates for phage therapy. They have a strictly lytic life cycle and a broad host range across *Staphylococci* (27). *Staphylococcus* bacteriophage genomes can be intentionally activated for engineering purposes through a couple of methods: transformation of L-form bacteria (28, 29) or using non-electroporation *Staphylococcus* transformation (30). These techniques enable the engineering of smaller phage genomes. However, like all synthetic methods, engineering becomes challenging when dealing with larger phage genomes that require assembly from numerous long DNA fragments. In addition, the transformation of *S. aureus* hosts is notoriously inefficient due to restriction-modification barriers, which prevent genome rebooting in more tractable heterologous systems such as *Escherichia coli*. Taken together, these factors currently preclude the use of synthetic biology-based strategies—such as whole-genome synthesis or yeast-based phage assembly—for large *S. aureus* phages. We therefore chose a homologous recombination (HR)-based approach, which has been successfully used for engineering larger phage genomes (19, 31–36), and included a downstream CRISPR-Cas-assisted counterselection (CS) step, which allowed us to rapidly obtain recombinant phages. Our goal was to adapt and validate this established approach for the first time in a strictly lytic *S. aureus* phage belonging to the *Twortvirinae* subfamily.

To demonstrate the functionality and applicability of this approach, we engineered a phage K-based reporter phage that enables the detection of viable *S. aureus* cells. Reporter phages are engineered to carry a heterologous reporter gene, such as a luciferase, which is expressed during infection. The expression of this gene generates a detectable signal, indicating the presence of viable host cells (37–39). Reporter phages offer significant advantages for bacterial detection, combining the speed and simplicity of PCR-based methods with the ability to detect viable cells. This contrasts with traditional culture-based methods, which, while reliable for detecting viable cells, are time-consuming and often require several days to yield results (40, 41).

Prior research has demonstrated the successful engineering of strictly lytic, *S. aureus*-infecting reporter bacteriophages through the use of HR (42). Notably, this work resulted in the positive identification of 97.7% of 390 clinical MRSA isolates at low bacterial concentrations. Recognizing the potential difficulties in the engineering process, our study aimed to devise a strategy that enables the generation of recombinant phages, even those that cannot be isolated through dilution enrichment due to the absence of a detectable reporter protein. This approach is particularly useful for systems characterized by low recombination rates, where traditional methods may prove labor-intensive and time-consuming. To address this, our engineering pipeline incorporates a CRISPR-Cas-based CS system, facilitating the isolation of recombinant phages following HR. The efficacy of this approach has been validated by its successful application in other phage-host systems (21, 43, 44, 44–50).

Our engineered reporter phage K::*nluc* was subjected to a comprehensive evaluation involving a diverse panel of 71 *S*. *aureus* strains, including clinical isolates with varying degrees of vancomycin resistance. Intriguingly, through the use of bioluminescence detection following treatment with K::*nluc*, our approach allowed for the identification of strains that displayed no signs of productive infection when analyzed using conventional plaque assays. Furthermore, we demonstrated the functionality of K::*nluc* in complex matrices, such as bovine raw milk and human whole blood. These findings underscore the potential of K::*nluc* for the rapid detection of *S. aureus* strains and suggest promising avenues for future research in diagnostic settings.

## RESULTS

### CRISPR-Cas9-assisted engineering of *Staphylococcus* reporter phage K

**Fig 1:**
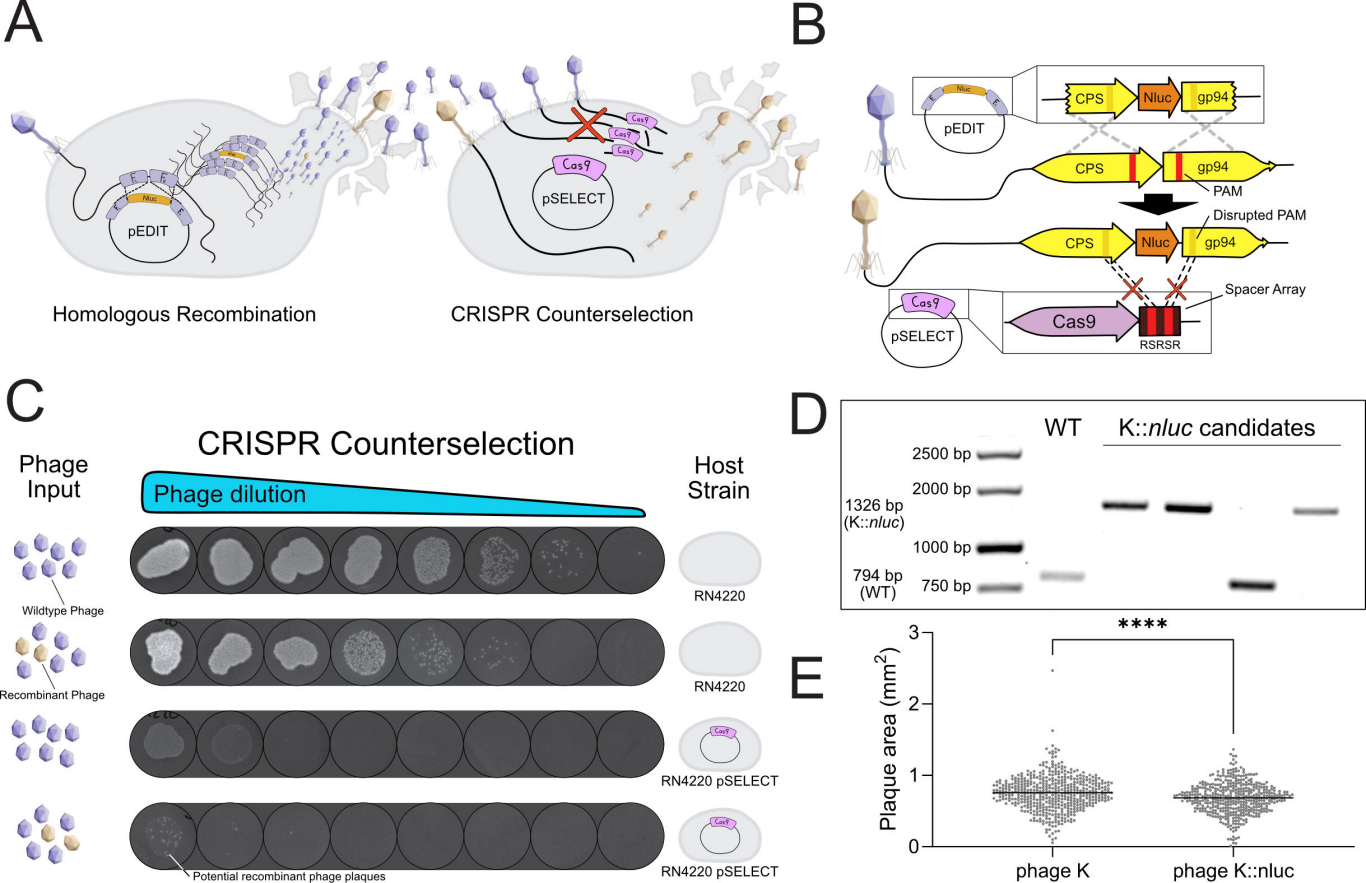
Construction of reporter phage K::*nluc* using a homologous recombination-based and CRISPR-Cas9-assisted phage engineering platform. (**A**) We employ two *S*. *aureus* bacterial host strains in our process. The first is the recombination donor, RN4220 pEDIT_nluc_ (RN4220 transformed with the HR donor plasmid pEDIT_nluc_), and the second is the counterselection strain, RN4220 pSELECT_CPS_. By sequentially infecting these host strains, we can generate (homologous recombination) and enrich (counterselection) the engineered phage. (**B**) The design of pEDIT_nluc_ and pSELECT_CPS_ facilitates selective amplification of phage particles that have undergone homologous recombination. This is made possible through two synonymous, single-nucleotide polymorphisms in the protospacer adjacent motifs (PAMs) of the donor template. The Cas9-mediated restriction of wild-type phage DNA is guided by two spacer sequences on pSELECT_CPS_, flanked by repeat regions (RS1RS2R), which target each homology arm on the target phage genome. (**C**) The efficiency of the CRISPR-Cas9 counterselection system is demonstrated. Wild-type lysate or recombination lysate was serially diluted and spotted on wild-type RN4220 (top two rows) or on the counterselection strain (bottom two rows). While wild-type phages were completely restricted (row three, limit of detection: 100 PFU/mL), visible plaques at the lowest dilutions in row four indicate the presence of phage variants that have escaped CRISPR-Cas9 restriction. (**D**) Individual plaques were selected, and the presence of an insertion of the intended size was validated using PCR. Positive PCR products were then Sanger sequenced to determine the correct genotype at the insertion site. (**E**) Quantitative analysis of plaque sizes for phage K and K::*nluc* on *S. aureus* PSK. Plaques were analyzed from scanned assay plates using ImageJ. A small but statistically significant reduction in plaque area was observed for K::*nluc* compared to wild-type phage K (Δ = −0.06985 mm^2^; *n* = 489 and 448 plaques, respectively; unpaired *t*-test, *P* < 0.0001).

We established a two-step protocol that includes HR and subsequent CS, as illustrated in Fig. 1A. In order to circumvent the pervasive restriction barriers often found in many *S. aureus* isolates, we opted to use *S. aureus* RN4220. This laboratory strain, obtained through extensive chemical mutagenesis of strain 8325-4, has had its resident prophages and restriction-modification systems removed. This modification allows for the transformation of RN4220 with *E. coli*-derived plasmid DNA (51).

Phage K, characterized by its broad host range, was selected as our engineering scaffold. Its host range primarily includes *S. aureus* and extends to other *Staphylococcus* species (52), making it a versatile candidate for both diagnostic and therapeutic purposes. This potential is evidenced by successful applications of *Twortvirinae* phages in therapeutic contexts, which have demonstrated their efficacy against a variety of clinically relevant bacterial strains (53–60).

As a proof of concept for our engineering pipeline, we constructed a bioluminescent reporter phage, K::*nluc*, by inserting the nanoluciferase (*nluc*) gene into the genome of phage K. Utilizing existing data on genome structure and transcriptomic profiles, we identified a region associated with the major capsid protein (*cps*) as a favorable locus for payload insertion. This region has been shown to be highly expressed in phage K (61) and has also served as a common integration site for payload insertions in other phages (21, 31, 42). To mediate expression from a strong, endogenous promoter, we integrated *nluc*—along with a ribosomal binding site—immediately downstream of the *cps* coding sequence. Nanoluciferase (NLuc), a bioluminescent luciferase derived from *Oplophorus gracilirostris* (NanoLuc, Promega), has been used successfully in prior phage engineering studies and enables sensitive detection due to its small size (516 bp) and strong luminescent output (42, 62).

The first step of the pipeline involves propagating phage K in the presence of the homology donor plasmid (pEDIT_nluc_). This plasmid carries the payload gene, along with up- and downstream flanking homology arms, which guide the sequence-specific integration of the payload.

The lysate of phage K, produced on RN4220 pEDIT_nluc_, comprises a heterogeneous population, predominantly of wild-type phages, interspersed with a minority of recombinants. This mixed lysate was subsequently propagated on another RN4220 host, one equipped with a CS plasmid (pSELECT_CPS_). This plasmid encodes an episomal CRISPR-Cas system, serving as the principal mechanism of selection to isolate the desired recombinant phages.

The pSELECT_CPS_ plasmid encodes the *S. pyogenes cas9* gene, along with tracrRNA and crRNA elements. The crRNA element carries a repeat-spacer1-repeat-spacer2-repeat (RS1RS2R) sequence, with each spacer targeting one homology arm flanking the *nluc* integration site. We designed the protospacer-adjacent motifs (PAMs) in the editing template (pEDIT_nluc_) to contain silent PAM mutations. This design enables K::*nluc* replication in the presence of pSELECT_CPS_ (Fig. 1B). Infection of RN4220 pSELECT_CPS_ with wild-type phage K resulted in complete phage restriction (Fig. 1C). When the same host strain was infected with a lysate containing a mixed population of recombinant and wild-type phages following HR, escape mutants were obtained at a frequency of approximately $10^{-4}$ (Fig. 1C). We then performed PCR amplification of the insertion site and found that three out of four plaques we picked yielded PCR products of the expected size (1,326 bp, Fig. 1D), indicating successful payload integration. Further confirmation was obtained through Sanger sequencing of the PCR products with the correct band size, which revealed a correct insertion of the payload at the intended locus.

### Characterization of K::*nluc* infectivity and bioluminescence kinetics across clinically relevant *S. aureus* strains

To assess whether reporter integration affected plaque morphology, we quantitatively compared plaque sizes of K::*nluc* and wild-type phage K on PSK using ImageJ-based analysis of plaque assay plates. This revealed a small but statistically significant reduction in plaque diameter for K::*nluc* (Fig. 1E). We then proceeded to assess the infectivity of K::*nluc* across a diverse set of 71 different *S. aureus* strains using efficiency of plaquing (EOP) assays (Fig. S1). This set included the phage propagation host PSK, common laboratory strains, and clinical isolates with varying degrees of vancomycin resistance. Out of 71 strains tested, 51 showed plaque formation (limit of detection: 100 PFU/mL) when treated with K::*nluc*, indicating successful infection by the phage. This assessment is crucial as it provides insight into the potential range of application for K::*nluc* in detecting different *S. aureus* strains.

Next, we established the minimum inhibitory concentration (MIC) of vancomycin for all strains, utilizing the cutoffs as defined by reference 63: vancomycin-susceptible (VSSA) strains exhibited an MIC ≤ 2 µg mL^−1^, vancomycin intermediate-resistant (VISA) strains showed an MIC between 4 and 8 µg mL^−1^, and vancomycin-resistant (VRSA) strains had an MIC ≥ 16 µg mL^−1^ (Fig. S2). We selected one phage K-susceptible isolate from each category (PSK [VSSA], LI6 [VISA], and VRSA7 [VRSA]), and investigated the kinetics of phage K::*nluc*-induced bioluminescence emission.

**Fig 2:**
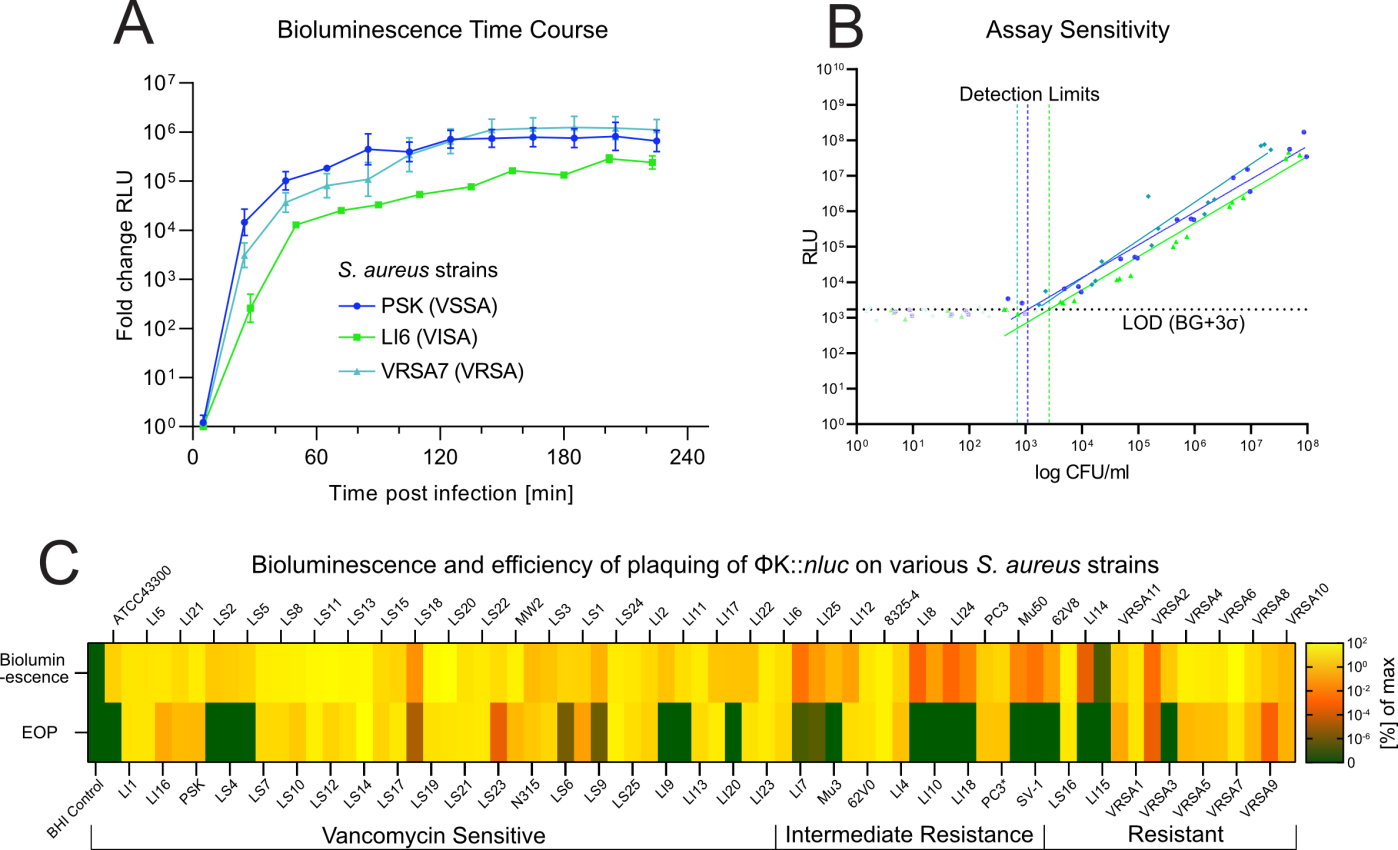
(**A**) Bioluminescence time course measurements for *S. aureus* strains PSK, LI6, and VRSA7. Fold-change in relative light units (RLU) was calculated by subtracting background luminescence (K::*nluc* in BHI alone) and normalizing to the signal from wild-type phage K infection of the same strain. Experiments were performed at a bacterial density of OD_600_ = 0.01 and a phage titer of 5 × 10^7^ PFU/mL. Data represent the mean ± standard deviation of biological replicates (*n* = 3). (**B**) Minimal dose response of PSK, LI6, and VRSA7 to K::*nluc* was determined by measuring the RLU after 3 h of infection for varying bacterial concentrations. The detection limits (vertical dotted lines) were calculated as the minimum cell number required to produce a signal that is higher than three standard deviations (σ) above the background luminescence (horizontal dotted lines). Measurements below this cutoff were excluded from linear regression. (**C**) Bioluminescence and efficiency of plaquing (EOP) for 71 *S*. *aureus* isolates. Bioluminescence for each strain is represented as the mean measured bioluminescence 3 h post-infection (*n* = 3). Values were background-corrected by subtracting luminescence from K::*nluc* in BHI alone. EOP is given by the mean number of plaque-forming units (PFU) after infection of a specific strain with K::*nluc* (three to six replicates per strain). Values are normalized to the host with the highest measurement (LS20 for bioluminescence, LS14 for EOP).

In all three K::*nluc* infections (PSK, LI6, and VRSA7), we observed similar signal intensities and kinetics of bioluminescence generation. There was a swift and consistent increase until it reached a plateau, with a peak fold-change of approximately 1 × 10^6^ relative light units (RLU) above the background luminescence, achieved roughly after 3 h (Fig. 2A).

To further assess the sensitivity of our reporter phage system, we examined the minimum dose response of bioluminescence emitted by PSK, LI6, and VRSA7 upon infection with a fixed concentration of K::*nluc* (5 × 10^7^ PFU/mL) (Fig. 2B). Although the minimum dose response of LI6 (2,617 CFU/mL) was higher than that of PSK (1,093 CFU/mL) and VRSA7 (707 CFU/mL), all values fall within the same order of magnitude. These results are in line with detection thresholds reported for other reporter phage systems (42, 62, 64).

Subsequently, we quantified the range of bioluminescence detection of K::*nluc* across all 71 *S*. *aureus* strains and compared it with the previously determined EOP (Fig. 2C). We successfully detected bioluminescence above background levels in all tested strains (71/71), encompassing even those 20 strains that had previously exhibited no evident plaque formation. To further assess the specificity of K::*nluc*, we also tested its activity against 17 non-*Staphylococcus* species and observed no bioluminescent signal indicative of active infection (Fig. S4).

### K::*nluc* detection of VSSA, VRSA, and VISA in human whole blood and bovine raw milk

*Staphylococcus* bacteremia accounts for a significant proportion of bloodstream infections (65, 66). Considering the complexity of blood as a biological matrix compared to standard laboratory culture conditions, we tested our reporter phage’s functionality in whole human blood. It is essential to evaluate this, as the *in vitro* infection kinetics of K::*nluc* with pathogenic bacterial strains might differ in more complex biological settings (67–69). We optimized the assay conditions using whole human blood spiked with the PSK strain, as depicted in Fig. S3. The most rapid and robust signal response was achieved under the following conditions: the spiked blood was diluted fivefold in growth medium, and the cells were incubated at 37°C for 1 h to stimulate host cell metabolism before reporter phage infection. We observed higher bioluminescent signals when using a citrate-based anticoagulant (Na3-citrate, citric acid, glucose, and potassium sorbate) compared to Li-Heparin, both of which are common storage solutions in clinical practice.

**Fig 3:**
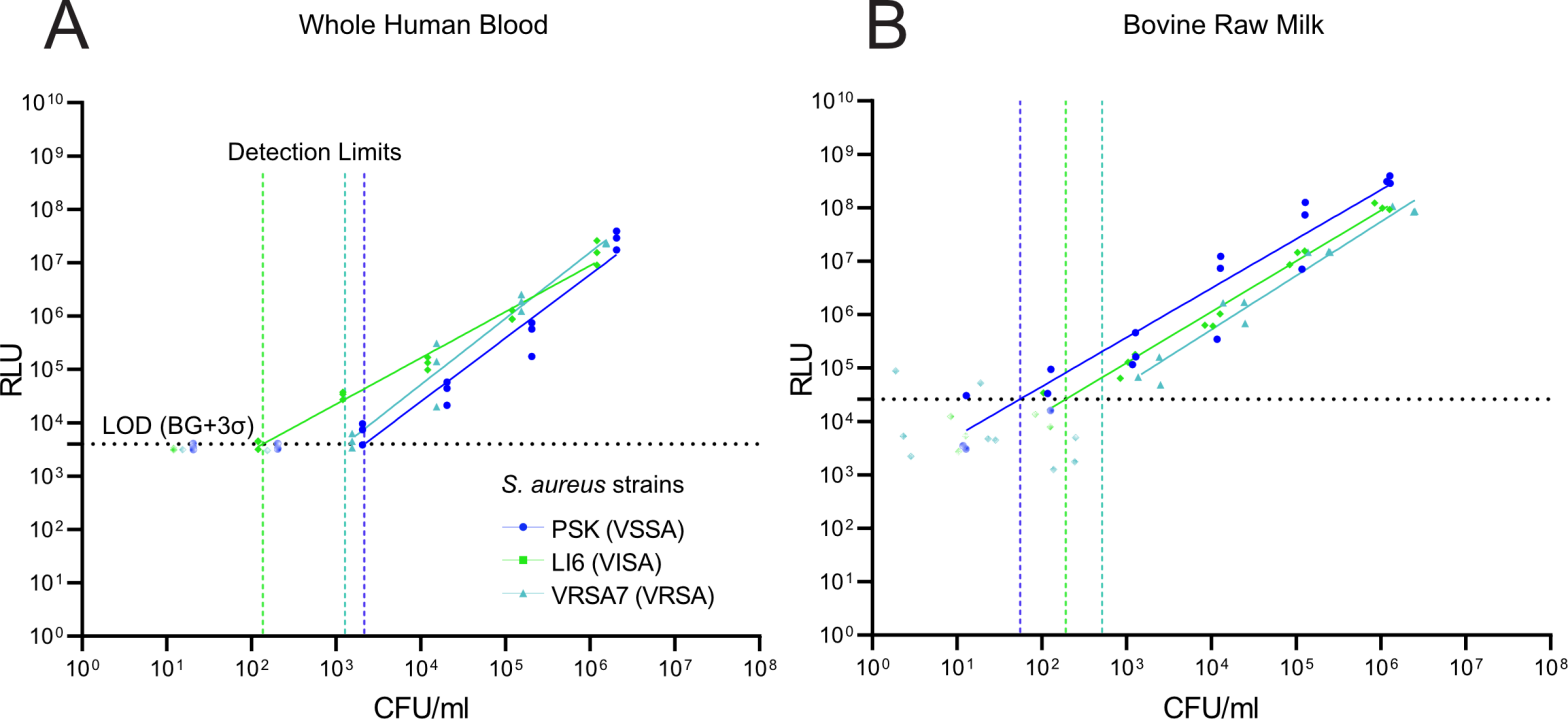
Detection of vancomycin-resistant *S. aureus* in whole human blood and bovine raw milk. Minimal dose response of PSK, LI6, and VRSA7 to K::*nluc* in whole human blood (**A**) and bovine raw milk (**B**) was determined by measuring the RLU after 3 h of infection for varying bacterial concentrations. Values below the determined limit of detection, set at 3 standard deviations of the mean background luminescence, were excluded. The detection limits (vertical dotted lines) were calculated as the minimum cell number required to produce a reliable signal (3 standard deviations [σ]) above the mean background luminescence (horizontal dotted line).

Leveraging these optimized conditions, we determined the detection limits using the previously selected *S. aureus* strains PSK, LI6, and VRSA7 (Fig. 3A). These strains were detectable at concentrations as low as 2,151 CFU/mL (PSK), 136 CFU/mL (LI6), and 1,270 CFU/mL (VRSA7). While there are minor differences in the detection thresholds across media, these values remain within 1 order of magnitude of those obtained in growth medium (Fig. 2B) and demonstrate robust detection in a clinically relevant matrix.

Bovine raw milk, another complex matrix, has also been reported to negatively impact bacteriophage proliferation (70–72). To investigate this, we conducted a similar experiment, replacing blood with unpasteurized, raw bovine milk. Contrary to expectations, treating bovine raw milk infected with *S. aureus* strains PSK, LI6, and VRSA7 with K::*nluc* resulted in bioluminescence detectable at minimal dose responses of 55 CFU/mL (PSK), 191 CFU/mL (LI6), and 514 CFU/mL (VRSA7). These results indicate that our assay performs reliably even in a matrix previously shown to inhibit phage activity, suggesting that the optimized protocol is robust to environmental complexity.

## DISCUSSION

In the face of rising antimicrobial resistance, bacteriophage therapy has garnered increasing attention as a potential alternative to traditional antibiotics (73). The engineering of bacteriophages has opened up new possibilities for diagnostic applications and enhancing clinical efficacy (12, 37). While there are numerous examples of engineered bacteriophages, the engineering of large, strictly lytic, *S. aureus*-infecting bacteriophages has been less explored. A previous study has successfully incorporated *nluc* into two *S*. *aureus*-infecting phages, ISP and MP115 (42). ISP and MP115 have been utilized in applications such as phage therapy and Food and Drug Administration (FDA)-approved KeyPath MRSA/methicillin-susceptible *S*. aureus assays, respectively (42, 74, 75). Our study made use of phage K, a member of the Kayvirus subfamily within the *Twortvirinae*, which is recognized for its well-characterized biology, including a transcriptional landscape analysis (61, 76). We developed a CRISPR-Cas-assisted engineering system for the rapid and reliable generation of genetically altered variants of phage K. As proof of concept, we utilized this pipeline to engineer a phage K mutant containing a bioluminescent *nluc* reporter gene, K::*nluc*.

Given the transformation barriers encountered with clinical *S. aureus* isolates, our current pipeline is constrained to phages capable of infecting restriction-deficient *Staphylococcus* strains. To engineer other phages, a host compatible with the transformation of pEDIT and pSELECT vectors is necessary. For bacteriophages that do not infect RN4220, our approach would necessitate finding a suitable, transformable host, potentially requiring additional steps to remove restriction-modification systems and resident prophages, akin to the modifications made to RN4220 (51, 77). An alternative approach is the cloning in dcm-*E. coli* strains that carry artificial modification systems (78). By acknowledging these challenges and implementing effective workarounds, we hope to broaden the applicability of our engineering strategy in the future.

Our engineering workflow was validated through the construction of K::*nluc*, a reporter gene-coding phage K variant for viable *S. aureus* detection. The bioluminescent nanoluciferase, NanoLuc, has been utilized in prior reporter phage development studies (42, 62, 79–81). Unlike antimicrobial payloads that disrupt key metabolic pathways and alter bacterium-phage dynamics, intracellular expression of NanoLuc is less likely to impose significant fitness costs on the phage or host bacterium. Thus, using *nluc* as a payload offered a simple benchmark for our engineering pipeline that was unlikely to affect the fitness of phage K. Given its large genome size (148 kb) and terminal redundancy, phage K tolerates the integration of the relatively small (516 bp) *nluc* payload without inducing genome packaging defects. We postulate that the integration of even larger payloads could be feasible, expanding the scope of potential applications, although a systematic analysis of such scenarios was not conducted in our study.

In standard spot-on-lawn infection screens, 20 out of 71 bacterial strains did not exhibit plaque formation of K::*nluc*, yet bioluminescence was detected in all strains. This observation suggests that phage binding, DNA delivery, and gene expression are still occurring, even in the absence of visible plaque formation. In such scenarios, it is likely that the infection cycle is interrupted—possibly by the cell undergoing abortive infection—before the completion of lysis and the release of progeny phage particles. For diagnostic applications, this implies that partially phage-resistant strains can be detected successfully as well, albeit with somewhat reduced sensitivity. At the same time, our data demonstrate that a single Kayvirus scaffold could be used to deliver therapeutic effector genes to most *S. aureus* isolates. The observed reduction in plaque size suggests a minor fitness cost associated with the insertion of the *nluc* reporter, which did, however, not affect the suitability of the reporter phage as a diagnostic tool.

The composition of blood can influence phage infection kinetics. At the same time, bacteriophages may be inactivated by innate or adaptive immune responses (67–69). Given these factors, it is imperative to acknowledge that the *in vitro* infection kinetics observed may not directly correlate to more complex biological environments. Thus, each reporter phage assay needs to be tailored to the specific matrix or application where a diagnostic need is identified. Within the scope of *S. aureus*, our focus is on bacteremia and bovine mastitis, representing pertinent diseases in humans and animals, respectively. For phage K, it has been previously demonstrated that the presence of whey proteins in bovine raw milk can competitively inhibit the phage’s attachment to the cell surface, consequently significantly diminishing infection efficiency (70, 72). Notably, in our experiments, there was no difference in observed detection limits. This may be attributed to matrix dilution, to the added 1 h activation step, or potentially to a low baseline concentration of whey proteins present in our milk sample.

While the potential of engineered bacteriophages in the treatment and diagnostics of *S. aureus* infections is well-acknowledged, their immediate implementation is hampered by several challenges. For instance, the swift progression of severe symptoms in cases of bacteremia, leading to sepsis, often mandates immediate intervention with broad-spectrum antibiotics, without preliminary identification of the causative pathogen. Nonetheless, the growing inclination toward patient-specific treatments and precision medicine is paving the way for the application of engineered phages. Specifically, our pipeline could facilitate the engineering of *S. aureus* phages to express antimicrobial effector genes, presenting a promising avenue for the future treatment of chronic infections such as wounds, pulmonary conditions, and implant infections, or as a last resort for bacteremia treatment when antibiotics are ineffective. In addition to therapeutic applications, engineered reporter phages such as K::*nluc* may also support diagnostic workflows. Given their ability to rapidly quantify viable bacterial cells, the use of K::*nluc* for antibiotic susceptibility testing (AST) represents an interesting avenue for future research, as has been demonstrated for *Klebsiella pneumoniae* (82).

Additionally, the broad infectivity of K::*nluc* provides significant advantages by enabling the detection of a wide range of strains. However, it also brings forth challenges that need thoughtful consideration, particularly the risk of false positive detections of other *Staphylococcus* species. *Staphylococcus epidermidis*, commonly found in human skin microbiota (83), serves as a prime example. Several strains of *S. epidermidis* have shown susceptibility to phage K infection, underscoring the importance of careful interpretation of detection results across diverse biological samples (52).

Finally, while the interaction of bacteriophages with dormant bacterial cells is complex and not fully understood, the potential for reporter phages to interact with such cells presents an intriguing area for future investigation. For *E. coli* and *P. aeruginosa*, there are instances where phages have been observed to adsorb to dormant hosts and deliver their genome, albeit subsequently entering a state of hibernation or pseudolysogeny, respectively (84, 85). Recent studies have also reported the phage infection and lysis of *P. aeruginosa* dormant persister cells (86). Therefore, future studies could explore the potential of K::*nluc* to interact with and potentially detect dormant *S. aureus* cells, contributing to a more comprehensive understanding of bacteriophage-host dynamics.

In summary, our study presents a novel and validated engineering pipeline for the generation of genetically altered phage K variants, offering promising avenues for *S. aureus* detection and treatment. While challenges remain in translating these findings to complex biological settings, the insights gained here lay the groundwork for further exploration and optimization, potentially contributing to the growing arsenal of tools in the fight against antimicrobial resistance.

## MATERIALS AND METHODS

### Vancomycin MIC of selected *S. aureus* strains

The MIC of the 71 *S*. *aureus* strains used in this study was determined under the standard culture conditions as outlined in reference 63. Briefly, 96-well plates were prepared, each well containing 250 µL of Miller-Hinton broth supplemented with a range of vancomycin concentrations (from 0.0625 to 64 µg/mL, with a twofold increase between subsequent concentrations). Each well was inoculated with 2 µL of 1:1,000 diluted bacterial cultures (grown overnight at 37°C) for each of the 71 strains. After an incubation period of 18 h at 37°C, turbidity was measured photometrically (OD_600_). Wells with an OD_600_ > 0.1 were considered turbid. The strains were then classified as VSSA (MIC ≤ 2 µg/mL), VISA (MIC = 4 to 8 µg/mL), or VRSA (MIC ≥ 16 µg/mL) based on the MIC values.

### Bacterial strains and culture conditions

*S. aureus* PSK (ATCC 19685) was used as propagation and *S. aureus* RN4220 (DSM 26309) as the engineering host of K and K::*nluc. E. coli* XL1-blue MRF’ (Stratagene) was used for plasmid amplification prior to RN4220 transformation. RN4220 and XL1-blue cultures were grown overnight (O/N) at 37°C in brain heart infusion (BHI) broth (Biolife Italiana) and Luria-Bertani/lysogeny broth (LB) medium (3 M sodium chloride, 10 g/L tryptone, and 5 g/L yeast extract, pH 7.2), respectively. The selection of 71 laboratory strains and clinical isolates (Fig. S2) were grown on BHI Broth with corresponding antibiotic supplements.

### Bacteriophage propagation

Phage K was propagated on *S. aureus* PSK using the soft-agar overlay method with BHI as bottom agar and LC agar (LB supplemented with 10 mM CaCl_2_, 10 mM MgSO_4_, and 10 g/L glucose) as top agar. Overlays were incubated O/N at 37°C and phages extracted from plates with semi-confluent lysis using 5 mL SM buffer (4°C, 2 h, constant agitation). Lysates were sterile-filtered (0.22 µm pores). Phage particles were precipitated O/N at 4°C using polyethylene glycol (7% PEG8000 and 1 M NaCl) and purified using stepped CsCl gradient ultracentrifugation. The obtained phage suspension was dialyzed twice against 1,000× excess of SM buffer. Purified samples were then stored long-term at 4°C. The titer was determined using the soft-agar overlay method.

### Electroporation of bacterial strains

XL1-blue electrocompetent cells were electroporated at 2.5 kV, 200 Ω, 25 µF, incubated for 1 h with SOC recovery medium ( 2% [wt/vol] Tryptone, 0.5% [wt/vol] yeast extract, 10 mM NaCl, 2.5 mM KCl, 10 mM MgCl_2_, and 20 mM glucose) at 37°C, and plated on selective agar to isolate successful transformants. RN4220 electrocompetent cells were electroporated at 1.8 kV, 600 Ω, 10 µF with500 to 1,000 ng of amplified plasmid DNA, incubated for 1 h at 37°C in B2 recovery medium (10 g/L casein hydrolysate, 5 g/L d-glucose, 1 g/L potassium phosphate dibasic, 25 g/L NaCl, and 25 g/L yeast extract) and plated on selective medium to isolate successful transformants.

### Plasmid design

The plasmid pLEB579 (kindly gifted by T. Takala, University of Helsinki, Finland) is a shuttle vector shown to have high transformation efficiencies in both *E. coli* and *S. aureus* and was therefore used as a backbone for both the editing template (pEDIT_nluc_) and the CRISPR-Cas9-counterselection system (pSELECT_CPS_). We used a previously reported, *Streptococcus pyogenes*-derived Cas9 (SpyCas9)-based CRISPR system (21) and exchanged the two spacers in the repeat-spacer1-repeat-spacer2-repeat (RS1RS2R) region. This was done to allow targeted restriction of wildtype phage K at two distinct loci (8,304 and 148 bp up- and downstream of the intended insertion site, respectively) designed to contain a PAM-disrupting synonymous mutation in the successful recombinants. pEDIT_nluc_ was constructed by integrating an nluc gene (optimized for *S. aureus* codon usage, avoidance of Rho-independent termination, and an added upstream ribosome binding site: GAGGAGGTAAATATAT), flanked by 400 bp (upstream) and 300 bp (downstream) homology arms, corresponding to the intended K insertion site, into the linearized pLEB579 backbone. Two silent point mutations were included to disrupt the two PAMs adjacent to the target DNA (Fig. 1B). All synthetic sequences were acquired as GeneArt String DNA Fragments (Thermo Fisher), albeit the RS1RS2R sequences, which were ordered as GeneArt Gene Synthesis (Thermo Fisher) (Table S2). Assembly of all constructs was performed using isothermal Gibson Assembly (22) (NEBuilder HiFi DNA Assembly Master Mix) and subsequently transformed into *E. coli* XL1-blue MRF’ for plasmid amplification. Plasmids and primers are listed in (Table S1).

### CRISPR-Cas9-assisted engineering of K::*nluc*

pEDIT_nluc_ and pSELECT_CPS_ were transformed into *S. aureus* RN4220 to acquire the strains required for recombination and counterselection, respectively. RN4220 pEDIT_nluc_ was infected with K via soft-agar overlay and a high titer lysate obtained as described previously (see Bacteriophage propagation section). Plates showing semi-confluent lysis were washed with SM buffer and 10-fold dilutions of the resulting lysate were used to perform soft-agar overlays on RN4220 pSELECT_CPS_. Individual plaques were picked from the plates showing the fewest (non-zero) plaques, resuspended in SM buffer, and clonally isolated by three rounds of plaque-purification. PCR amplification using primers flanking the insert site (Table S1) was performed and products showing a size indicative of the intended insertion were purified and Sanger sequenced (Microsynth AG, Balgach, Switzerland) to validate the correct genomic sequence. Validated phage lysates were purified using ultracentrifugation on a cesium-chloride gradient and subsequent dialysis as described in Bacteriophage propagation section.

### Soft-agar overlay

BHI soft agar (5 mL) was melted and cooled to 47°C. The molten soft-agar was inoculated with 200 µL bacterial culture of adequate turbidity (OD_600_> 1) and 10 µL of phage suspension, briefly mixed by agitation, and spread evenly onto BHI agar plates with. Plates were let dry for 15 min at room temperature (RT) and subsequently inverted and incubated at 37°C for 12–18 h.

### Spot-on-lawn assay

BHI soft agar (5 mL) was melted and cooled to 47°C. The molten soft-agar was inoculated with 200 µL of bacterial culture, briefly mixed by vortexing, and spread evenly on BHI agar plates. Plates were dried at RT for 15 min; droplets of phage suspension were then placed carefully on the dried soft agar. Plates were dried for 15 min, inverted, and incubated at 37°C for 16 h.

### Determination of EOP

EOP of bacteriophage suspensions was determined by performing spot-on-lawn assays on bacterial strains of interest. Tenfold dilutions of the phage suspension were prepared up to a maximum dilution of 1 × 10^−8^. Spot-on-lawn assays were performed as described in the previous section using the series of bacteriophage dilutions and O/N cultures of the corresponding host strains. The EOP is given by the number of plaque-forming units (PFU) after infection of a specific strain with K::*nluc*. Values are relative to the host with the highest measurement (LS20 for bioluminescence, LS14 for EOP).

### Plaque size quantification

To assess whether reporter gene insertion affected phage fitness, we compared plaque sizes between wild-type phage K and K::*nluc*. Both phages were plated at similar titers on three independent *S. aureus* PSK cultures using 1/2 BHI agar plates and LC soft agar. Plates were incubated overnight at 37°C and scanned at 1,000 dpi. Image analysis was performed in Fiji/ImageJ using a custom macro. All images were processed with identical brightness settings and subjected to automatic thresholding. Plaques with an area larger than 10 pixels and circularity above 0.75 were included. Manual cleanup was used to remove overlapping or irregular plaques. Mean plaque areas were compared using an unpaired two-tailed *t*-test in GraphPad Prism.

### Bioluminescence time course assay

Stationary phase bacterial cultures were diluted to OD_600_ = 0.01, inoculated with 5 × 10^7^ PFU/mL K::*nluc,* and incubated at 37°C (180 rpm agitation). Bioluminescence measurements were taken by combining 25 µL of the sample solution with an equal volume of prepared buffer-reconstituted nluc substrate as detailed by the manufacturer (NanoGlo Luciferase Assay System; Promega). Measurements were taken every 20 min (225 min total) in Nunc F96 MicroWell 446 plates using a GloMax navigator luminometer (Promega) with 5 s integration time and 2 s delay. To account for background luminescence from residual Nanoluciferase in the purified phage preparation, all K::*nluc* infection measurements were first background corrected by subtracting the signal obtained from a phage-only control (K::*nluc* in BHI medium). To calculate fold-change values, background-corrected RLU values were subsequently divided by the corresponding RLU values obtained from a wild-type phage K control infection on the same strain. This approach yields a background-corrected fold-change of RLU.

### Minimal dose-response

To determine the minimum concentration of cells giving a significant bioluminescence signal above the background, stationary phase bacterial cultures were diluted to a range of concentrations in ten-fold increments (OD_600_ = 10^−1^ to 10^−10^). Measurements were taken 3 h post-infection (5 × 10^7^ PFU/mL). The minimum concentration of cells that could be detected was established as the smallest measurement exceeding a signal threshold, defined as the mean plus three times the standard deviation of the background signal. The theoretical minimal concentration was ascertained at the point where the established signal threshold intersects with a linear regression of the data, considering only RLU values surpassing the threshold.

### Determination of bioluminescence for the 71 *S. aureus* strains used in the study

Stationary phase bacterial cultures were diluted to OD_600_ = 0.01, inoculated with 5 × 10^7^ PFU/mL K::*nluc*, and incubated at 37°C with 180 rpm agitation. Bioluminescence was measured at 3 h post-infection and background corrected by subtracting the signal obtained from K::*nluc* incubated in BHI alone. For each strain, the mean RLU value was determined from three biological replicates. Background-corrected RLU values were then normalized to the highest measured response (strain LS20) to allow for comparison across strains.

### K::*nluc*-based detection of *S. aureus* in patient blood and bovine raw milk

Minimal dose response of K::*nluc* infection on one representative each of VSSA, VISA, and VRSA was conducted in triplicate as done with regular growth medium described above, albeit with some modifications. First, spiked whole human blood or bovine raw milk was mixed 1:5 with BHI growth medium and incubated for 1 h at 37°C with agitation (180 rpm) prior to infection with 5 × 10^7^ PFU/mL K::*nluc*. Bioluminescence was measured after 3 h. The whole human blood samples were stored in anticoagulant solutions containing either 1.89 mg/mL Na3-citrate, 0.69 mg/mL citric acid, 2.1 mg/mL glucose, and 0.03 mg/mL potassium sorbate (BD Vacutainer (REF 367756), Becton, Dickinson and Company, NJ, USA) or Li-Heparin (17 IU/mL) (BD Vacutainer (REF 368886), Becton, Dickinson and Company).

### Software

CLC Genomics Workbench version 20.0.4 was used for sequence analyses such as primer and string design as well as evaluation of Sanger sequencing results. Plotting was done using GraphPad Prism version 10.0.0 for Windows. OpenAI’s ChatGPT 4 (87) was used as a tool to assist with formatting and editing the manuscript. This involved iterative refinements to ensure clarity and conciseness of the content presented.

## Data Availability

All raw data, plasmids, and the reporter phage are available upon request. The plasmid sequences of pSELECTCPS and pEDITnluc are available in GenBank under accession numbers PV779750 and PV779751, respectively.

## SUPPLEMENTAL MATERIAL

Supplemental material aem.02014-24-s0001.pdf Figures S1 to S3; Tables S1 and S2.

ASM does not own the copyrights to Supplemental Material that may be linked to, or accessed through, an article. The authors have granted ASM a non-exclusive, world-wide license to publish the Supplemental Material files. Please contact the corresponding author directly for reuse.

## References

[1] Aslam B, Wang W, Arshad MI, Khurshid M, Muzammil S, Rasool MH, Nisar MA, Alvi RF, Aslam MA, Qamar MU, Salamat MKF, Baloch Z. 2018. Antibiotic resistance: a rundown of a global crisis. Infect Drug Resist 11:1645–1658. doi:10.2147/IDR.S173867 30349322 PMC6188119

[2] De Oliveira DMP, Forde BM, Kidd TJ, Harris PNA, Schembri MA, Beatson SA, Paterson DL, Walker MJ. 2020. Antimicrobial resistance in ESKAPE pathogens. Clin Microbiol Rev 33:10–1128. doi:10.1128/CMR.00181-19 PMC7227449 32404435

[3] Prestinaci F, Pezzotti P, Pantosti A. 2015. Antimicrobial resistance: a global multifaceted phenomenon. Pathog Glob Health 109:309–318. doi:10.1179/2047773215Y.0000000030 26343252 PMC4768623

[4] Aslam B, Khurshid M, Arshad MI, Muzammil S, Rasool M, Yasmeen N, Shah T, Chaudhry TH, Rasool MH, Shahid A, Xueshan X, Baloch Z. 2021. Antibiotic resistance: one health one world outlook. Front Cell Infect Microbiol 11:771510. doi:10.3389/fcimb.2021.771510 34900756 PMC8656695

[5] O’neill J. 2014. Antimicrobial resistance: tackling a crisis for the health and wealth of nations. Review on Antimicrobial Resistance

[6] Perry J, Waglechner N, Wright G. 2016. The prehistory of antibiotic resistance. Cold Spring Harb Perspect Med 6:a025197. doi:10.1101/cshperspect.a025197 27252395 PMC4888810

[7] Skandalis N, Maeusli M, Papafotis D, Miller S, Lee B, Theologidis I, Luna B. 2021. Environmental spread of antibiotic resistance. Antibiotics (Basel) 10:640. doi:10.3390/antibiotics10060640 34071771 PMC8226744

[8] Hatfull GF, Dedrick RM, Schooley RT. 2022. Phage therapy for antibiotic resistant bacterial infections. Annu Rev Med 73:197–211. doi:10.1146/annurev-med-080219-122208 34428079

[9] Anyaegbunam NJ, Anekpo CC, Anyaegbunam ZKG, Doowuese Y, Chinaka CB, Odo OJ, Sharndama HC, Okeke OP, Mba IE. 2022. The resurgence of phage-based therapy in the era of increasing antibiotic resistance: from research progress to challenges and prospects. Microbiol Res 264:127155. doi:10.1016/j.micres.2022.127155 35969943

[10] McCallin S, Sacher JC, Zheng J, Chan BK. 2019. Current state of compassionate phage therapy. Viruses 11:343. doi:10.3390/v11040343 31013833 PMC6521059

[11] Hitchcock NM, Devequi Gomes Nunes D, Shiach J, Valeria Saraiva Hodel K, Dantas Viana Barbosa J, Alencar Pereira Rodrigues L, Coler BS, Botelho Pereira Soares M, Badaró R. 2023. Current clinical landscape and global potential of bacteriophage therapy. Viruses 15:1020. doi:10.3390/v15041020 37113000 PMC10146840

[12] Lenneman BR, Fernbach J, Loessner MJ, Lu TK, Kilcher S. 2021. Enhancing phage therapy through synthetic biology and genome engineering. Curr Opin Biotechnol 68:151–159. doi:10.1016/j.copbio.2020.11.003 33310655 PMC11996084

[13] Meile S, Du J, Dunne M, Kilcher S, Loessner MJ. 2022. Engineering therapeutic phages for enhanced antibacterial efficacy. Curr Opin Virol 52:182–191. doi:10.1016/j.coviro.2021.12.003 34952266

[14] Mahler M, Costa AR, van Beljouw SPB, Fineran PC, Brouns SJJ. 2023. Approaches for bacteriophage genome engineering. Trends Biotechnol 41:669–685. doi:10.1016/j.tibtech.2022.08.008 36117025

[15] Usman SS, Uba AI, Christina E. 2023. Bacteriophage genome engineering for phage therapy to combat bacterial antimicrobial resistance as an alternative to antibiotics. Mol Biol Rep 50:7055–7067. doi:10.1007/s11033-023-08557-4 37392288

[16] Tétart F, Repoila F, Monod C, Krisch HM. 1996. Bacteriophage T4 host range is expanded by duplications of a small domain of the tail fiber adhesin. J Mol Biol 258:726–731. doi:10.1006/jmbi.1996.0281 8637004

[17] Dunne M, Rupf B, Tala M, Qabrati X, Ernst P, Shen Y, Sumrall E, Heeb L, Plückthun A, Loessner MJ, Kilcher S. 2019. Reprogramming bacteriophage host range through structure guided design of chimeric receptor binding proteins. Cell Rep 29:1336–1350. doi:10.1016/j.celrep.2019.09.062 31665644

[18] Yehl K, Lemire S, Yang AC, Ando H, Mimee M, Torres MDT, de la Fuente-Nunez C, Lu TK. 2019. Engineering phage host-range and suppressing bacterial resistance through phage tail fiber mutagenesis. Cell 179:459–469. doi:10.1016/j.cell.2019.09.015 31585083 PMC6924272

[19] Zhang J, Ning H, Lin H, She J, Wang L, Jing Y, Wang J. 2022. Expansion of the plaquing host range and improvement of the absorption rate of a T5-like Salmonella phage by altering the long tail fibers. Appl Environ Microbiol 88. doi:10.1128/aem.00895-22 PMC9469705 35969059

[20] Dedrick RM, Guerrero-Bustamante CA, Garlena RA, Russell DA, Ford K, Harris K, Gilmour KC, Soothill J, Jacobs-Sera D, Schooley RT, Hatfull GF, Spencer H. 2019. Engineered bacteriophages for treatment of a patient with a disseminated drug-resistant Mycobacterium abscessus. Nat Med 25:730–733. doi:10.1038/s41591-019-0437-z 31068712 PMC6557439

[21] Du J, Meile S, Baggenstos J, Jäggi T, Piffaretti P, Hunold L, Matter CI, Leitner L, Kessler TM, Loessner MJ, Kilcher S, Dunne M. 2023. Enhancing bacteriophage therapeutics through in situ production and release of heterologous antimicrobial effectors. Nat Commun 14:4337. doi:10.1038/s41467-023-39612-0 37474516 PMC10359290

[22] Gibson DG, Young L, Chuang R-Y, Venter JC, Hutchison CA 3rd, Smith HO. 2009. Enzymatic assembly of DNA molecules up to several hundred kilobases. Nat Methods 6:343–345. doi:10.1038/nmeth.1318 19363495

[23] Jaschke PR, Lieberman EK, Rodriguez J, Sierra A, Endy D. 2012. A fully decompressed synthetic bacteriophage øX174 genome assembled and archived in yeast. Virology (Auckl) 434:278–284. doi:10.1016/j.virol.2012.09.020 23079106

[24] Cheung GY, Bae JS, Otto M. 2021. Pathogenicity and virulence of Staphylococcus aureus Virulence 12:547–569. doi:10.1080/21505594.2021.1878688 33522395 PMC7872022

[25] van Hal SJ, Jensen SO, Vaska VL, Espedido BA, Paterson DL, Gosbell IB. 2012. Predictors of mortality in Staphylococcus aureus bacteremia. Clin Microbiol Rev 25:362–386. doi:10.1128/CMR.05022-11 22491776 PMC3346297

[26] Centers for disease control and prevention. 2021. 2019 Antibiotic resistance threats report. Available from: https://www.cdc.gov/drugresistance/biggest-threats.html

[27] Vandersteegen K, Kropinski AM, Nash JH, Noben J-P, Hermans K, Lavigne R. 2013. Romulus and Remus, two phage isolates representing a distinct clade within the Twortlikevirus genus, display suitable properties for phage therapy applications. J Virol 87:3237–3247. doi:10.1128/JVI.02763-12 23302893 PMC3592175

[28] Kilcher S, Studer P, Muessner C, Klumpp J, Loessner MJ. 2018. Cross-genus rebooting of custom-made, synthetic bacteriophage genomes in L-form bacteria. Proc Natl Acad Sci USA 115:567–572. doi:10.1073/pnas.1714658115 29298913 PMC5776983

[29] Fernbach J, Meile S, Kilcher S, Loessner MJ. 2023. Genetic engineering and rebooting of bacteriophages in L-form bacteria, p 247–259. In Bacteriophage therapy: from lab to clinical practice. Springer. 10.1007/978-1-0716-3523-0_16 38066374

[30] Assad-Garcia N, D’Souza R, Buzzeo R, Tripathi A, Oldfield LM, Vashee S, Fouts DE. 2022. Cross-genus “boot-up” of synthetic bacteriophage in Staphylococcus aureus by using a new and efficient DNA Transformation method. Appl Environ Microbiol 88:e0148621. doi:10.1128/AEM.01486-21 34818102 PMC8824277

[31] Loessner MJ, Rees CE, Stewart GS, Scherer S. 1996. Construction of luciferase reporter bacteriophage A511::luxAB for rapid and sensitive detection of viable Listeria cells. Appl Environ Microbiol 62:1133–1140. doi:10.1128/aem.62.4.1133-1140.1996 8919773 PMC167878

[32] Namura M, Hijikata T, Miyanaga K, Tanji Y. 2008. Detection of Escherichia coli with fluorescent labeled phages that have a broad host range to E. coli in sewage water. Biotechnol Prog 24:481–486. doi:10.1021/bp070326c 18225914

[33] Mahichi F, Synnott AJ, Yamamichi K, Osada T, Tanji Y. 2009. Site-specific recombination of T2 phage using IP008 long tail fiber genes provides a targeted method for expanding host range while retaining lytic activity. FEMS Microbiol Lett 295:211–217. doi:10.1111/j.1574-6968.2009.01588.x 19453513

[34] Erickson S, Paulson J, Brown M, Hahn W, Gil J, Barron-Montenegro R, Moreno-Switt AI, Eisenberg M, Nguyen MM. 2021. Isolation and engineering of a Listeria grayi bacteriophage. Sci Rep 11:18947. doi:10.1038/s41598-021-98134-1 34556683 PMC8460666

[35] Shitrit D, Hackl T, Laurenceau R, Raho N, Carlson MC, Sabehi G, Schwartz DA, Chisholm SW, Lindell D. 2022. Genetic engineering of marine cyanophages reveals integration but not lysogeny in T7-like cyanophages. ISME J 16:488–499. doi:10.1038/s41396-021-01085-8 34429521 PMC8776855

[36] Guan J, Oromí-Bosch A, Mendoza SD, Karambelkar S, Berry JD, Bondy-Denomy J. 2022. Bacteriophage genome engineering with CRISPR–Cas13a. Nat Microbiol 7:1956–1966. doi:10.1038/s41564-022-01243-4 36316452 PMC9722621

[37] Meile S, Kilcher S, Loessner MJ, Dunne M. 2020. Reporter phage-based detection of bacterial pathogens: design guidelines and recent developments. Viruses 12:944. doi:10.3390/v12090944 32858938 PMC7552063

[38] Hussain W, Ullah MW, Farooq U, Aziz A, Wang S. 2021. Bacteriophage-based advanced bacterial detection: concept, mechanisms, and applications. Biosens Bioelectron 177:112973. doi:10.1016/j.bios.2021.112973 33429203

[39] Ye J, Guo J, Li T, Tian J, Yu M, Wang X, Majeed U, Song W, Xiao J, Luo Y, Yue T. 2022. Phage‐based technologies for highly sensitive luminescent detection of foodborne pathogens and microbial toxins: a review. Comp Rev Food Sci Food Safe 21:1843–1867. doi:10.1111/1541-4337.12908 35142431

[40] Peri AM, Harris PN, Paterson DL. 2022. Culture-independent detection systems for bloodstream infection. Clin Microbiol Infect 28:195–201. doi:10.1016/j.cmi.2021.09.039 34687856

[41] Foddai AC, Grant IR. 2020. Methods for detection of viable foodborne pathogens: current state-of-art and future prospects. Appl Microbiol Biotechnol 104:4281–4288. doi:10.1007/s00253-020-10542-x 32215710 PMC7190587

[42] Brown M, Hahn W, Bailey B, Hall A, Rodriguez G, Zahn H, Eisenberg M, Erickson S. 2020. Development and evaluation of a sensitive bacteriophage-based MRSA diagnostic screen. Viruses 12:631. doi:10.3390/v12060631 32545159 PMC7354448

[43] Kiro R, Shitrit D, Qimron U. 2014. Efficient engineering of a bacteriophage genome using the type I-E CRISPR-Cas system. RNA Biol 11:42–44. doi:10.4161/rna.27766 24457913 PMC3929423

[44] Tao P, Wu X, Tang W-C, Zhu J, Rao V. 2017. Engineering of bacteriophage T4 genome using CRISPR-Cas9. ACS Synth Biol 6:1952–1961. doi:10.1021/acssynbio.7b00179 28657724 PMC5771229

[45] Manor M, Qimron U. 2017. Selection of genetically modified bacteriophages using the CRISPR-Cas system. Bio Protoc 7:e2431. doi:10.21769/BioProtoc.2431 PMC5553622 28804739

[46] Møller-Olsen C, Ho SFS, Shukla RD, Feher T, Sagona AP. 2018. Engineered K1F bacteriophages kill intracellular Escherichia coli K1 in human epithelial cells. Sci Rep 8:17559. doi:10.1038/s41598-018-35859-6 30510202 PMC6277420

[47] Hupfeld M, Trasanidou D, Ramazzini L, Klumpp J, Loessner MJ, Kilcher S. 2018. A functional type II-A CRISPR-Cas system from Listeria enables efficient genome editing of large non-integrating bacteriophage. Nucleic Acids Res 46:6920–6933. doi:10.1093/nar/gky544 30053228 PMC6061871

[48] Corts AD, Thomason LC, Gill RT, Gralnick JA. 2019. Efficient and precise genome editing in Shewanella with recombineering and CRISPR/Cas9-mediated counter-selection. ACS Synth Biol 8:1877–1889. doi:10.1021/acssynbio.9b00188 31277550

[49] Lemay M-L, Tremblay DM, Moineau S. 2017. Genome engineering of virulent lactococcal phages using CRISPR-Cas9. ACS Synth Biol 6:1351–1358. doi:10.1021/acssynbio.6b00388 28324650

[50] Duong MM, Carmody CM, Ma Q, Peters JE, Nugen SR. 2020. Optimization of T4 phage engineering via CRISPR/Cas9. Sci Rep 10:18229. doi:10.1038/s41598-020-75426-6 33106580 PMC7588440

[51] Kreiswirth BN, Löfdahl S, Betley MJ, O’Reilly M, Schlievert PM, Bergdoll MS, Novick RP. 1983. The toxic shock syndrome exotoxin structural gene is not detectably transmitted by a prophage. Nature 305:709–712. doi:10.1038/305709a0 6226876

[52] Göller PC, Elsener T, Lorgé D, Radulovic N, Bernardi V, Naumann A, Amri N, Khatchatourova E, Coutinho FH, Loessner MJ, Gómez-Sanz E. 2021. Multi-species host range of staphylococcal phages isolated from wastewater. Nat Commun 12:6965. doi:10.1038/s41467-021-27037-6 34845206 PMC8629997

[53] O’Flaherty S, Ross RP, Meaney W, Fitzgerald GF, Elbreki MF, Coffey A. 2005. Potential of the polyvalent anti-Staphylococcus bacteriophage K for control of antibiotic-resistant staphylococci from hospitals. Appl Environ Microbiol 71:1836–1842. doi:10.1128/AEM.71.4.1836-1842.2005 15812009 PMC1082512

[54] Merabishvili M, Pirnay J-P, Verbeken G, Chanishvili N, Tediashvili M, Lashkhi N, Glonti T, Krylov V, Mast J, Van Parys L, Lavigne R, Volckaert G, Mattheus W, Verween G, De Corte P, Rose T, Jennes S, Zizi M, De Vos D, Vaneechoutte M. 2009. Quality-controlled small-scale production of a well-defined bacteriophage cocktail for use in human clinical trials. PLoS ONE 4:e4944. doi:10.1371/journal.pone.0004944 19300511 PMC2654153

[55] Lehman SM, Mearns G, Rankin D, Cole RA, Smrekar F, Branston SD, Morales S. 2019. Design and preclinical development of a phage product for the treatment of antibiotic-resistant Staphylococcus aureus infections. Viruses 11:88. doi:10.3390/v11010088 30669652 PMC6356596

[56] Łubowska N, Grygorcewicz B, Kosznik-Kwaśnicka K, Zauszkiewicz-Pawlak A, Węgrzyn A, Dołęgowska B, Piechowicz L. 2019. Characterization of the three new kayviruses and their lytic activity against multidrug-resistant Staphylococcus aureus Microorganisms 7:471. doi:10.3390/microorganisms7100471 31635437 PMC6843549

[57] Van Nieuwenhuyse B, Galant C, Brichard B, Docquier P-L, Djebara S, Pirnay J-P, Van der Linden D, Merabishvili M, Chatzis O. 2021. A case of in situ phage therapy against Staphylococcus aureus in a bone allograft polymicrobial biofilm infection: outcomes and phage antibiotic interactions. Viruses 13:1898. doi:10.3390/v13101898 34696328 PMC8539586

[58] Kolenda C, Medina M, Legendre T, Blazere L, Bergot M, Arnaud V, Souche A, Roussel-Gaillard T, Martins-Simoes P, Tristan A, Ferry T, Laurent F. 2021. Development of phage therapy to treat staphylococci bone and joint infections in france: isolation and characterization of 17 novel anti-*Staphylococcus* bacteriophages, p 86–86. In Orthopaedic proceedings. Vol. 103. Bone and Joint.

[59] Onsea J, Post V, Buchholz T, Schwegler H, Zeiter S, Wagemans J, Pirnay J-P, Merabishvili M, D’Este M, Rotman SG, Trampuz A, Verhofstad MHJ, Obremskey WT, Lavigne R, Richards RG, Moriarty TF, Metsemakers W-J. 2021. Bacteriophage therapy for the prevention and treatment of fracture related infection caused by Staphylococcus aureus: a preclinical study. Microbiol Spectr 9:e0173621. doi:10.1128/spectrum.01736-21 34908439 PMC8672900

[60] Plumet L, Ahmad-Mansour N, Dunyach-Remy C, Kissa K, Sotto A, Lavigne J-P, Costechareyre D, Molle V. 2022. Bacteriophage therapy for Staphylococcus aureus infections: a review of animal models, treatments, and clinical trials. Front Cell Infect Microbiol 12:907314. doi:10.3389/fcimb.2022.907314 35782148 PMC9247187

[61] Finstrlová A, Mašlaňová I, Blasdel Reuter BG, Doškař J, Götz F, Pantůček R. 2022. Global transcriptomic analysis of bacteriophage-host interac tions between a Kayvirus therapeutic phage and Staphylococcus aureus. Microbiol Spectr 10:e0012322. doi:10.1128/spectrum.00123-22 35435752 PMC9241854

[62] Meile S, Sarbach A, Du J, Schuppler M, Saez C, Loessner MJ, Kilcher S. 2020. Engineered reporter phages for rapid bioluminescence-based detec tion and differentiation of viable Listeria cells. Appl Environ Microbiol 86:e00442-20. doi:10.1128/AEM.00442-20 32245761 PMC7237785

[63] Clinical and Laboratory Standards Institute. 2018. Performance standards for antimicrobial susceptibility testing. 28th ed. CLSI Supplement M100. Clinical and Laboratory Standards Institute, Wayne, PA.

[64] Kim S, Kim M, Ryu S. 2014. Development of an engineered bioluminescent reporter phage for the sensitive detection of viable Salmonella typhimurium. Anal Chem 86:5858–5864. doi:10.1021/ac500645c 24806327

[65] Reimer LG, Wilson ML, Weinstein MP. 1997. Update on detection of bacteremia and fungemia. Clin Microbiol Rev 10:444–465. doi:10.1128/CMR.10.3.444 9227861 PMC172929

[66] Viscoli C. 2016. Bloodstream Infections: the peak of the iceberg. Virulence 7:248–251. doi:10.1080/21505594.2016.1152440 26890622 PMC4871637

[67] Srivastava AS, Kaido T, Carrier E. 2004. Immunological factors that affect the in vivo fate of T7 phage in the mouse. J Virol Methods 115:99–104. doi:10.1016/j.jviromet.2003.09.009 14656466

[68] Principi N, Silvestri E, Esposito S. 2019. Advantages and limitations of bacterio phages for the treatment of bacterial infections. Front Pharmacol 10:513. doi:10.3389/fphar.2019.00513 31139086 PMC6517696

[69] Porayath C, Salim A, Palillam Veedu A, Babu P, Nair B, Madhavan A, Pal S. 2018. Characterization of the bacteriophages binding to human matrix molecules. Int J Biol Macromol 110:608–615. doi:10.1016/j.ijbiomac.2017.12.052 29246876 PMC5864510

[70] O’Flaherty S, Coffey A, Meaney WJ, Fitzgerald GF, Ross RP. 2005. Inhibition of bacteriophage K proliferation on Staphylococcus aureus in raw bovine milk. Lett Appl Microbiol 41:274–279. doi:10.1111/j.1472-765X.2005.01762.x 16108920

[71] Gill JJ, Pacan JC, Carson ME, Leslie KE, Griffiths MW, Sabour PM. 2006. Efficacy and pharmacokinetics of bacteriophage therapy in treatment of subclinical Staphylococcus aureus mastitis in lactating dairy cattle. Antimicrob Agents Chemother 50:2912–2918. doi:10.1128/AAC.01630-05 16940081 PMC1563511

[72] Gill JJ, Sabour PM, Leslie KE, Griffiths MW. 2006. Bovine whey proteins inhibit the interaction of Staphylococcus aureus and bacteriophage K. J Appl Microbiol 101:377–386. doi:10.1111/j.1365-2672.2006.02918.x 16882145

[73] Abedon ST, García P, Mullany P, Aminov R. 2017. Phage therapy: past, present and future. Front Microbiol 8:981. doi:10.3389/fmicb.2017.00981 28663740 PMC5471325

[74] Vandersteegen K, Mattheus W, Ceyssens P-J, Bilocq F, De Vos D, Pirnay J-P, Noben J-P, Merabishvili M, Lipinska U, Hermans K, Lavigne R. 2011. Microbiological and molecular assessment of bacteriophage ISP for the control of Staphylococcus aureus. PLoS One 6:e24418. doi:10.1371/journal.pone.0024418 21931710 PMC3170307

[75] Bhowmick T, Mirrett S, Reller LB, Price C, Qi C, Weinstein MP, Kirn TJ. 2013. Controlled multicenter evaluation of a bacteriophage-based method for rapid detection of Staphylococcus aureus in positive blood cultures. J Clin Microbiol 51:1226–1230. doi:10.1128/JCM.02967-12 23390282 PMC3666813

[76] Barylski J, Kropinski AM, Alikhan N-F, Adriaenssens EM, ICTV Report Consortium. 2020. ICTV virus taxonomy profile: Herelleviridae. J Gen Virol 101:362–363. doi:10.1099/jgv.0.001392 32022658 PMC7414437

[77] Nair D, Memmi G, Hernandez D, Bard J, Beaume M, Gill S, Francois P, Cheung AL. 2011. Whole-genome sequencing of Staphylococcus aureus strain RN4220, a key laboratory strain used in virulence research, identifies mutations that affect not only virulence factors but also the fitness of the strain. J Bacteriol 193:2332–2335. doi:10.1128/JB.00027-11 21378186 PMC3133102

[78] Jones MJ, Donegan NP, Mikheyeva IV, Cheung AL. 2015. Improving transformation of Staphylococcus aureus belonging to the CC1, CC5 and CC8 clonal complexes. PLoS One 10:e0119487. doi:10.1371/journal.pone.0119487 25807379 PMC4373697

[79] Zhang D, Coronel-Aguilera CP, Romero PL, Perry L, Minocha U, Rosenfield C, Gehring AG, Paoli GC, Bhunia AK, Applegate B. 2016. The use of a novel NanoLuc-based reporter phage for the detection of Escherichia coli O157: H7. Sci Rep 6. doi:10.1038/srep33235 PMC5021930 27624517

[80] Pulkkinen EM, Hinkley TC, Nugen SR. 2019. Utilizing in vitro DNA assembly to engineer a synthetic T7 Nanoluc reporter phage for Escherichia coli detection. Integr Biol (Camb) 11:63–68. doi:10.1093/intbio/zyz005 30927414

[81] Jain P, Garing S, Verma D, Saranathan R, Clute-Reinig N, Gadwa J, Peterson C, Hermansky G, Astashkina Fernandez A, Asare E, Weisbrod TR, Spencer E, Mulholland CV, Berney M, Bell D, Nichols KP, Le Ny A-LM, Ordway D, Jacobs WR Jr, Somoskovi A, Minch KJ. 2020. Nanoluciferase reporter mycobacteriophage for sensitive and rapid detection of Mycobacterium tuberculosis drug susceptibility. J Bacteriol 202:10–1128. doi:10.1128/JB.00411-20 PMC7585058 32900827

[82] Braun P, Raab R, Bugert JJ, Braun S. 2023. Recombinant reporter phage rTUN1::nLuc enables rapid detection and real-time antibiotic susceptibility testing of Klebsiella pneumoniae K64 strains. ACS Sens 8:630–639. doi:10.1021/acssensors.2c01822 36719711 PMC9972469

[83] Chen YE, Fischbach MA, Belkaid Y. 2018. Skin microbiota-host interactions. Nature 553:427–436. doi:10.1038/nature25177 29364286 PMC6075667

[84] Bryan D, El-Shibiny A, Hobbs Z, Porter J, Kutter EM. 2016. Bacteriophage T4 infection of stationary phase E. coli: life after log from a phage perspective. Front Microbiol 7:1391. doi:10.3389/fmicb.2016.01391 27660625 PMC5014867

[85] Ripp S, Miller RV. 1998. Dynamics of the pseudolysogenic response in slowly growing cells of Pseudomonas aeruginosa. Microbiology (Reading) 144 (Pt 8):2225–2232. doi:10.1099/00221287-144-8-2225 9720044

[86] Maffei E, Woischnig A-K, Burkolter MR, Heyer Y, Humolli D, Thürkauf N, Bock T, Schmidt A, Manfredi P, Egli A, Khanna N, Jenal U, Harms A. 2024. Phage paride can kill dormant, antibiotic-tolerant cells of Pseudomonas aeruginosa by direct lytic replication. Nat Commun 15:175. doi:10.1038/s41467-023-44157-3 38168031 PMC10761892

[87] OpenAI. 2023. GPT-4 technical report. arXiv. doi:10.48550/arXiv.2303.08774

