# Supplementary material for: CRISPR-Cas9 enables efficient genome engineering of the strictly lytic, broad-host-range staphylococcal bacteriophage K
Source: Appl Environ Microbiol. 2025 Aug 4;91(9):e02014-24. doi: 10.1128/aem.02014-24 (PMC12442396; doi:10.1128/aem.02014-24)
Supplement: Supplemental material — Figures S1 to S3; Tables S1 and S2. [file aem.02014-24-s0001.pdf]

Supplementary Material

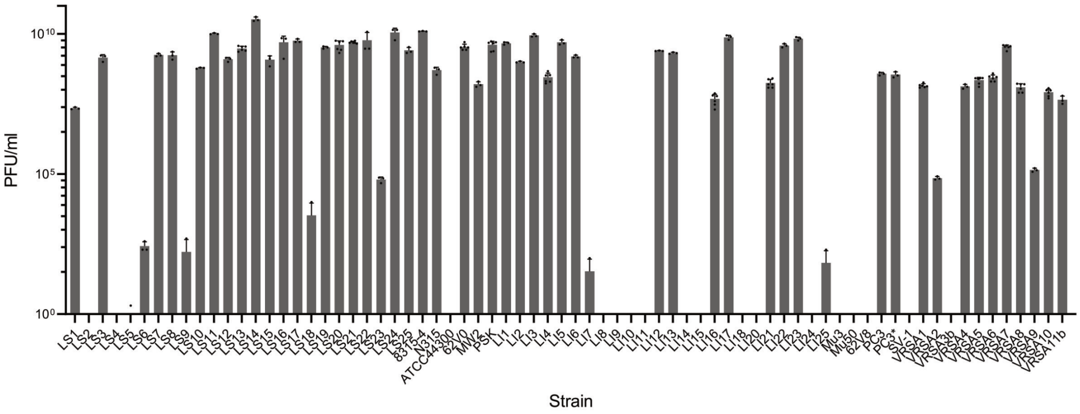

Supplementary Figure 1: Efficiency of plating (EOP) of K::*nluc* on a selection of 71 *S. aureus* species

Standard spot-on-lawn plaque assay was performed to elucidate the absolute EOP of K::*nluc* on a panel of 71 *S. aureus* species given by plaque forming units per volume (PFU/ml). Standard deviation was calculated from 3-6 replicates per strain.

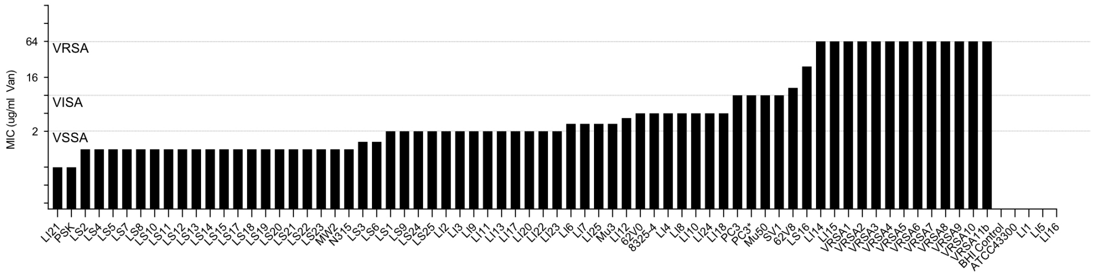

Supplementary Figure 2: Minimal inhibitory concentration (MIC) of vancomycin on bacterial strains used in this study

The minimum inhibitory concentration (MIC) of vancomycin on the 71 bacterial strain used in this study was tested by culturing in the presence of varying vancomycin concentrations, ranging from 0.0625 µg/mL – 64 µg/mL with twofold increase between subsequent concentrations. For cases where replicates did not yield uniform results, the MIC was determined as the triplicate mean. Vancomycin suceptibility (VSSA), intermediate resistance (VISA) and full resistance (VRSA) are indicated along with the MIC cutoffs defined by [1].

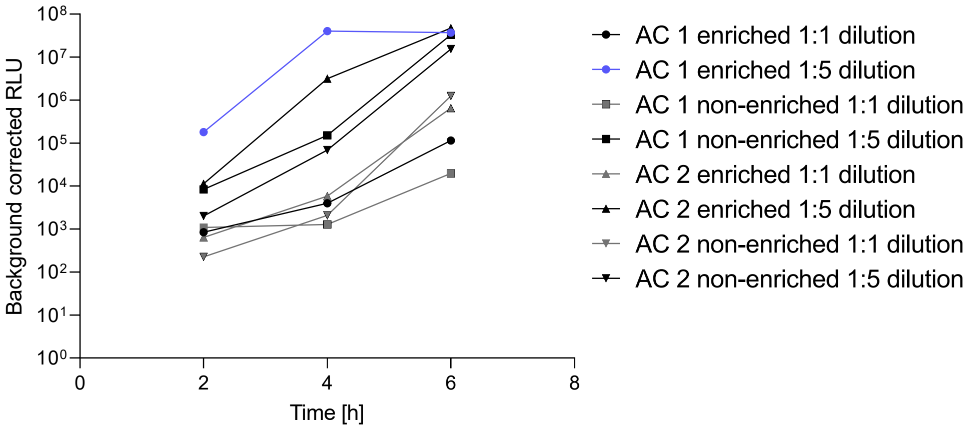

Supplementary Figure 3: Effects of various parameters on K::*nluc* infection-associated bioluminescence in human whole blood.

Whole human blood was spiked with different bacterial concentrations of *S. aureus* PSK. Samples were subsequently diluted with BHI growth medium at a ratio of 1:1 or 1:5. Samples were then either infected with 5×10^7 PFU/ml K::*nluc* or first incubated at 37 °C for 1 h prior to infection with K::*nluc*. These parameters were furthermore tested on two types of anticoagulant solutions, AC1 (Na3-citrate, citric acid, glucose, potassium sorbate) and AC2 (Li-Heparin). The combination of parameters resulting in the highest bioluminescence is highlighted in blue.

Supplementary Table 1: Primers and templates used for plasmid construction and insert confirmations.

Fragment	Template	Primer 1	P1 Sequence [5' -> 3']	Primer 2	P2 Sequence [5' -> 3']	Amplicon Size (bp)
pLEB579 SpyCas9 Backbone	pLEB579 SpyCas9 T4*	pLEB579 SpyCas9 Backbone Fw	GACTCCATTCAACATTGCCGA	pLEB579 SpyCas9 Backbone Rev	TCAGCTAGACTTCAGTCTTG AAAAG	7903
pLEB579 SpyCas9 K Spacers	Phage K Spacers Synthetic DNA String	Phage K Spacers Fw	GCAGTAATACAGGGGCTTTT C	Phage K Spacers Rev	CCTCTTTCTCAAGTTATCATC GG	350
pLEB579 Backbone	pLEB579	pLEB579 Backbone Fw	AGTCGATGTTAAACCGTGTG CTCTACG	pLEB579 Backbone Rev	CGCGCTATTAATCGCAACAT CAAACC	2848
pLEB579 ϕK_nluc_CPS Insert	Phage K Nanoluc Post Capsid Synthetic DNA String	Phage K Nluc HR Insert Fw	GGGGCTTTTATTTTGGTTTGA TGTTG	Phage K Nluc HR Insert Rev	TTATAGTTTTGGTCGTAGAG CACACG	1312
pEDITϕK_nluc _CPS Confirmation	pEDITϕK_nluc_C PS	pEDIT Confirmation Fw	GAGAAATGGAAGTTGAATTA AG	pEDIT Confirmatio n Rev	GATAATGAACTGTGCTGATT AC	1616
Phage K::nluc Confirmation	Phage K::nluc	Phage K::nluc Confirmation Fw	AGTAGTAGTTAACTCAGATG AC	Phage K::nluc Confirmatio n Rev	TGTAGATTTTCTAGTAGTAT TTGTAG	1326

* From [2].

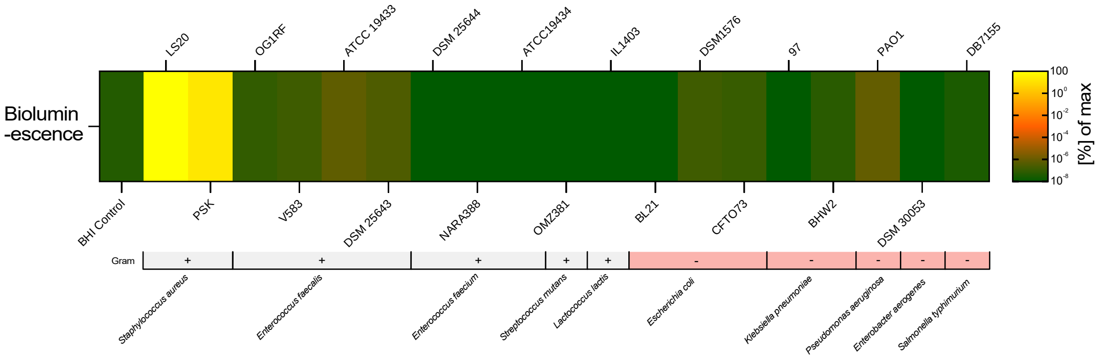

Supplementary Figure 4: Supplementary Figure S4: *K::nluc* reporter phage exhibits high specificity toward *Staphylococcus aureus*.

To evaluate off-target activity, *K::nluc* was tested against a panel of 17 non-*Staphylococcus* species, including Gram-positive and Gram-negative bacteria. Gram-positive strains included *Enterococcus faecalis* (OG1RF, V583, ATCC 19433, DSM 25643), *Enterococcus faecium* (DSM 25644, NARA388, ATCC 19434), *Streptococcus mutans* (OMZ381) and *Lactococcus lactis* (IL1403). Gram-negative species included *Escherichia coli* (BL21, DSM 1576, CFTO73), *Klebsiella pneumoniae* (97, BHW2), *Pseudomonas aeruginosa* (PAO1), *Enterobacter aerogenes* (DSM 30053) and *Salmonella typhimurium* (DB7155). Bioluminescence was measured 3 h after infection with *K::nluc* at a fixed phage concentration of 5×10^7 PFU/mL. Values were background-corrected using the signal from *K::nluc* incubated without bacteria (*K::nluc* + BHI), and are shown relative to the highest responding *S. aureus* host strain (LS20). PSK and LS20 were included as internal *S. aureus* controls.

Supplementary Table 2: SpyCas9 Phage K spacer string and pEDIT Phage K::*nluc* string used in this study.

**Synthetic String Name**	**Sequence [5' -> 3']**
SpyCas9 Phage K Spacer String	AGTATATTTTAGATGAAGATTATTTCTTAATAACTAAAAATATGGTATAATACTCTTAATAAATGCAGTAAT<br>ACAGGGGCTTTTCAAGACTGAAGTCTAGCTGAGACAAATAGTGCGATTACGAAATTTTTTAGACAAAAATA<br>GTCTACGAGGTTTTAGAGCTATGCTGTTTTGAATGGTCCCAAAACAGGAAGTACATACCTGTTTCTTTACCT<br>TGAGTTTTAGAGCTATGCTGTTTTGAATGGTCCCAAAACATAAAAAAATGGCTACTGTTTATGGTACAGGT<br>TTTAGAGCTATGCTGTTTTGAATGGTCCCAAAACTTCAGCACACTGAGACTTGTTGAGTTCCATGTTTTAGA<br>GCTATGCTGTTTTGAATGGACTCCATTCAACATTGCCGATGATAACTTGAGAAAGAGGGTTAATACCAGCA<br>GTCGGATACCTTCCTATTCTTTCTGTTAAAGCGTTTTCATGTTATAATAGGCAAAAGAAGAGTAGTGTGATC<br>GTCCATTCCGACAGCATCGCCAGTCACTAT
pEDIT Phage K Nanoluc String	AAAGTCGAAGGGGGCTTTTATTTTGGTTTGATGTTGCGATTAATAGCGCGAGTAACAGCTACAGTATCTAA<br>CGTAGACGATGGTGTTAAACTTTCAATTAATGTTAACGCTATGTACCAACAACAACCACAATTCGTTTCTAT<br>CTATCGTCAAGGTAAAGAAACAGGTATGTACTTCCTAATCAAACGTGTACCAGTTAAAGATGCACAAGAA<br>GACGGAACAATCGTATTCGTAGATAAGAACGAAACATTGCCTGAAACAGCAGACGTATTTGTTGGTGAAA<br>TGTCACCACAAGTAGTTCACTTATTCGAATTACTTCCAATGATGAAATTACCATTAGCTCAAATTAATGCTTC<br>TATTACATTTGCAGTATTATGGTATGGTGCATTAGCATTACGTGCTCCTAAAAAATGGGCTCGTATTAAAAA<br>CGTTCGTTATATCGCAGTTTAAGAGGAGGTAAATATATATGGTATTCACTTTAGAAGATTTCGTAGGTGAT<br>TGGCGTCAAACTGCTGGTTACAACTTAGATCAAGTATTAGAACAAGGTGGTGTATCATCATTATTCCAAAA<br>CTTAGGTGTATCAGTAACTCCAATCCAACGTATCGTATTATCAGGTGAAAACGGTTTAAAAATCGATATCC<br>ACGTAATCATCCCATACGAAGGTTTATCAGGTGATCAAATGGGTCAAATCGAAAAAATCTTCAAAGTAGTA<br>TACCCAGTAGATGATCACCACTTCAAAGTAATCTTACACTACGGTACTTTAGTAATCGATGGTGTAACTCCA<br>AACATGATCGATTACTTCGGTCGTCCATACGAAGGTATCGCTGTATTCGATGGTAAAAAAATCACTGTAAC<br>TGGTACTTTATGGAACGGTAACAAAATCATCGATGAACGTTTAATCAACCCAGATGGTTCATTATTATTCCG<br>TGTAACTATCAACGGTGTAACTGGTTGGCGTTTATGTGAACGTATCTTAGCTTAATAGAATAAGAAAAACT<br>GAATACAAGAGAATAGGGATAAACTTAGGGTTTATCCCTTTTTTATTAAAATAAACTTGAAGGGATTTAAT<br>AAATATGTTATACTATAAGAAACTATTAGATAAAAAAATGGCTACTGTTTATGGTACAGTTGAGATTGACA<br>AAGATGGAGTAGTCAAAGGATTAACTAAAGAACAAGAAAAAGAATTTGCCAATGTTCCAGGTTTTGAATT<br>TGAAGAAGAAAAGAAAACTACTAGAAAACAATCAGCTTCTACTAGTAAAGAAGAAGAGCCTAAGGAAGA<br>GGAAGTCGATGTTAAACCGTGTGCTCTACGACCAAAACTATAAAACCTTTAAG

References

1. Clinical & Institute, L. S. *Performance Standards for Antimicrobial Susceptibility Testing* 28th ed. (Clinical and Laboratory Standards Institute, Wayne, PA, 2018).
2. Meile, S., Du, J., Dunne, M., Kilcher, S. & Loessner, M. J. Engineering therapeutic phages for enhanced antibacterial efficacy. *Current opinion in virology* **52,** 182–191 (2022).
